# Supplementary material for: D-serine released by astrocytes in brainstem regulates breathing response to CO2 levels
Source: Nat Commun. 2017 Oct 10;8:838. doi: 10.1038/s41467-017-00960-3 (PMC5635109; doi:10.1038/s41467-017-00960-3)
Supplement: Supplementary file 1 — Supplementary information [file 41467_2017_960_MOESM1_ESM.pdf]

## Supplementary material

# D-serine released by astrocytes in the brainstem regulates breathing response to $CO_2$ levels

**Authors:** S. Beltrán-Castillo [1], M. J. Olivares[1], R.A. Contreras[1], G. Zúñiga[1], I. Llona[1], R. von Bernhardi[2*], and J. L. Eugenín[1*]

## Supplementary Figures

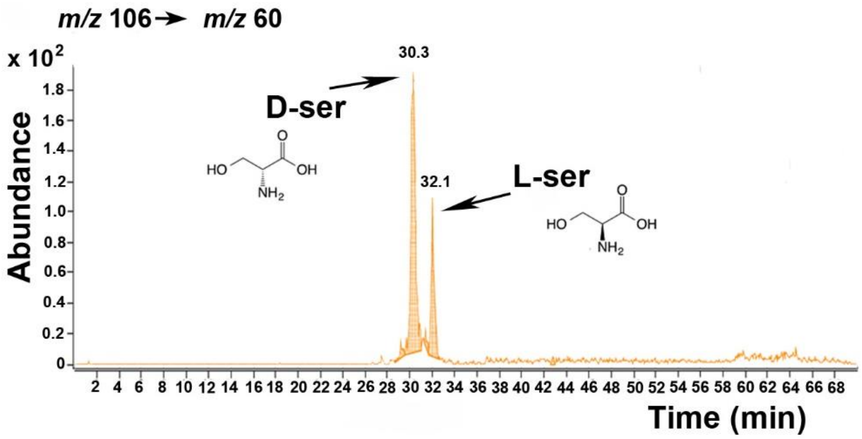


**Supplementary Fig. 1**. **LC-MS/MS chromatogram shows distinct peaks for D- and L-serine**. The detected peaks correspond to a mixture of D-serine (145 nM) and L-serine (37 nM) in aCSF. The retention times for D- and L- serine were 30.3 and 32.1 min, respectively. *m/z* corresponds to mass-to-charge ratio of the precursor and product ion, respectively.

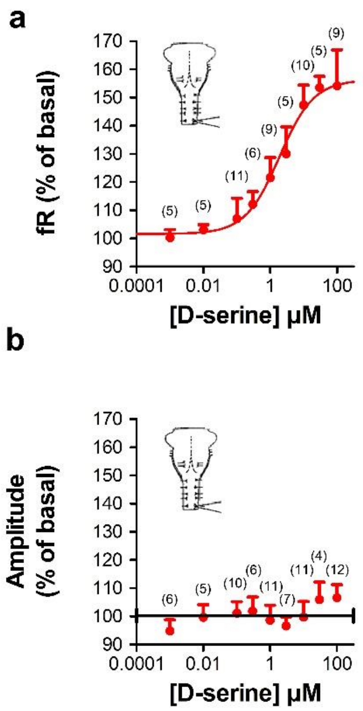


**Supplementary Fig. 2. D-serine increases fR in *en bloc* preparations**.

Concentration-response curves for the changes in fR (**a**) and amplitude (**b**) of fictive respiration induced by superfusion of aCSF with different concentrations of D-serine**.** The fR and the amplitude are expressed as percentage of respective basal value; symbols and vertical lines indicate means and SEM, respectively; numbers of independent preparations are indicated at the top of symbols. Horizontal line in (**b**) indicates the lack of change respect to basal values (100%). Insets show schemata of the *en bloc* preparation.

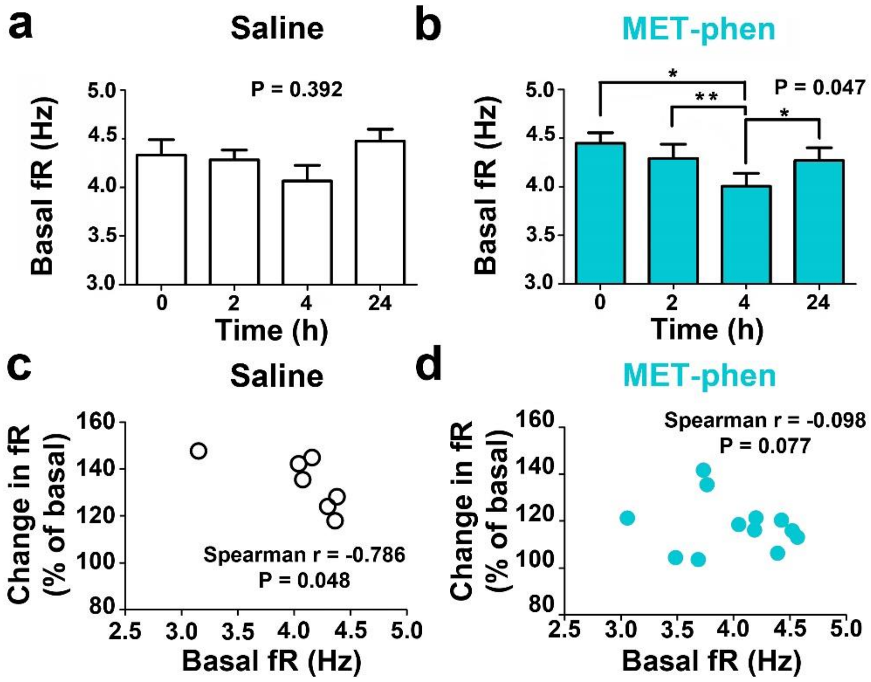


**Supplementary Fig. 3. Reduction in the fR response to hypercapnia after i.p. injection of MET-phen is not correlated with the reduction of the basal fR**. **(a,b)** The basal fR in conscious unrestrained adult mice after an i.p. injection of saline alone (**a**, controls, n = 6) or saline with 9 mg $kg^{-1}$ MET-phen (**b**, MET-phen, n = 12). (**c,d**) Correlations between the basal fR and the changes in fR induced by hypercapnia in controls (**c**) and MET-phen-treated (**d**) mice. There was a statistically significant negative correlation in control mice. Those mice showing the largest responses to hypercapnia were those showing the lowest basal fR. In MET-phen injected mice, no significant correlation was detected.

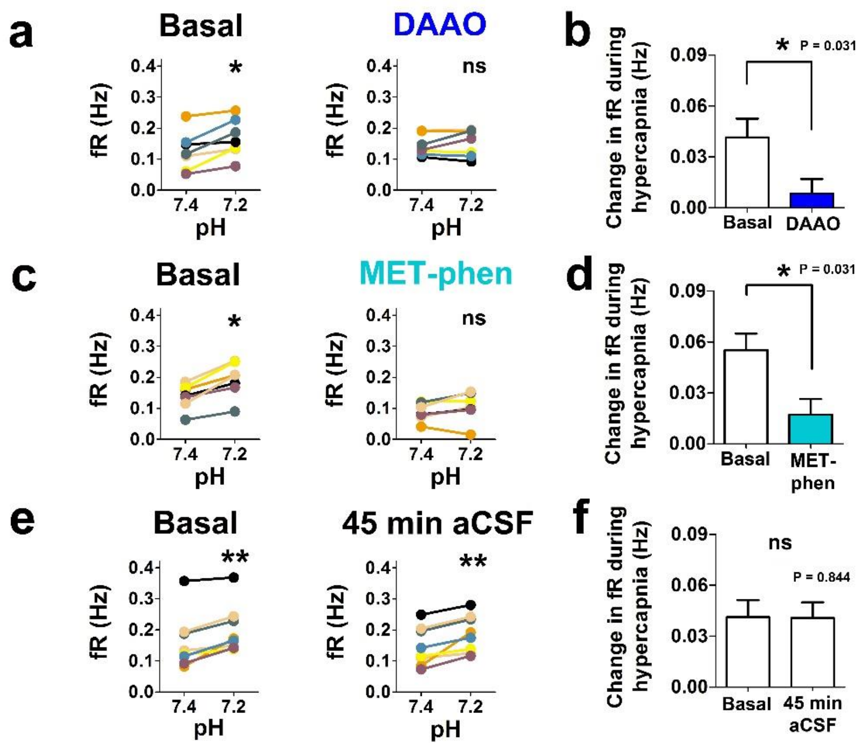


**Supplementary Fig. 4. The increase in fR induced by hypercapnic acidosis in *en bloc* preparations was reduced by D-serine degradation or D-serine racemase inhibition**. fR in *en bloc* preparations superfused with aCSF at pH 7.4 and 7.2 (hypercapnic acidosis) before (basal) and after 45 min of exposure to 0.1 U ml$^{-1}$ DAAO + 260 U ml$^{-1}$ catalase (**a**) or 50 µM MET-phen (**c**). Colored lines connect the fR values at pH 7.4 and 7.2 for each *en bloc* preparation. (**b,d**) Average changes in fR induced by hypercapnia before exposure (basal, white bars) and exposure to DAAO (**b,** blue bar) or MET-phen (**d,** cyan bar) for 45 min. **(e)** Changes in fR induced by hypercapnia at the beginning (basal) and after a 45 min period in which *en bloc* preparations were superfused only with aCSF. Colored lines connect the fR values at pH 7.4 and 7.2 for each *en bloc* preparation. **(f)** Average changes in fR induced by hypercapnia at the beginning and after 45 min of aCSF superfusion. Note that changes in fR induced by hypercapnic acidosis were reduced by DAAO and MET-phen treatments, but not by the aCSF superfusion period. Data are expressed as the mean ± SEM; *, and ** indicate P<0.05, and P<0.01, respectively (Wilcoxon signed rank test).

.

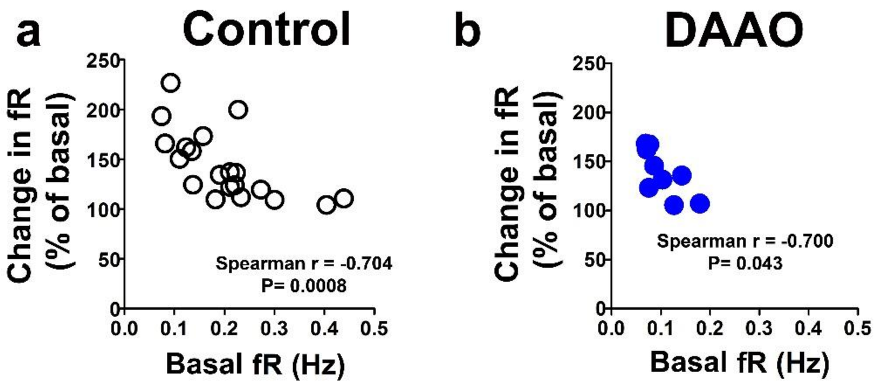


**Supplementary Fig. 5. Reduction in the fR response to hypercapnic acidosis observed with DAAO is not correlated with the reduction in the basal fR in caudal medullary brainstem slices**. The Spearman correlation test showed a negative correlation between the magnitude of fR response to hypercapnic acidosis and the baseline fR in control (**a**) and DAAO treated (**b**) slices. Thus, in both experimental conditions, those slices showing the largest fR responses to hypercapnic acidosis were those showing the lowest basal fR.

# Supplementary Table

**Supplementary Table 1. MRM ionization patterns for amino acids.** *L* and *D* amino acids exhibited the same ionization pattern, but the retention times differed according to the chirality properties.

| Amino acid | Precursor ion | Product ion | Fragmentor | Collision energy |
|---|---|---|---|---|
| Glu | 148 | 130 | 158 | 22 |
| Ser | 106 | 60 | 131 | 20 |
