# Supplementary material for: D-serine released by astrocytes in brainstem regulates breathing response to CO2 levels
Source: Nat Commun. 2017 Oct 10;8:838. doi: 10.1038/s41467-017-00960-3 (PMC5635109; doi:10.1038/s41467-017-00960-3)
Supplement: Supplementary file 3 — Description of Additional Supplementary Files [file 41467_2017_960_MOESM3_ESM.pdf]

Description of Additional Supplementary Files

File Name: Supplementary Data 1
Description: Individual/raw data for each figure and main text. This file was deposited also in https://figshare.com/ doi: 10.6084/m9.figshare.5239831
